# Gourmand-Like Syndrome With Sugar Preference Following Parieto-Occipital Metastasis: A Neuropsychiatric Case Report

**DOI:** 10.1192/j.eurpsy.2026.11621

**Published:** 2026-07-31

**Authors:** A. Bansal, P. Verma, M. Gupta

**Affiliations:** Department of Psychiatry, Vardhman Mahavir Medical College and Safdarjung Hospital, New Delhi, India

## Abstract

**Introduction:**

Gourmand Syndrome is a rare neuropsychiatric condition characterized by new-onset, intense preoccupation with specific foods, often sweets, following focal brain lesions involving the right anterior insula, orbitofrontal cortex, or their connections (Regard & Landis, Eur Neurol 1997;37:236–240). While most reported cases follow right-hemisphere involvement, mass effect from contralateral lesions may produce similar behavioral changes (Mendez et al., J Neuropsychiatry Clin Neurosci 2007;19:444–449). We present a dementia case in which persistent sugar craving and selective food aversion emerged after left parieto-occipital metastasis with mass effect on right-hemispheric reward circuits.

**Objectives:**

To present a rare case of late-life dementia with prominent sugar craving and food selectivity, occurring after a left parieto-occipital metastasis exerting mass effect on right-hemispheric gustatory–reward circuits.

**Methods:**

An observational case study was conducted, integrating neuroimaging findings, clinical psychiatric evaluation, caregiver reports, and behavioral analysis over a three-month follow-up. MRI brain scans before and after stereotactic radiotherapy were reviewed to assess lesion evolution and anatomical involvement

**Results:**

An 89-year-old male with no psychiatric history, with adenocarcinoma treated in 2021 (4 years back), developed seizures and subsequent cognitive decline following hyponatremia. Neuroimaging revealed a left occipital metastasis with edema and progressive midline shift toward the right hemisphere. Post-radiotherapy, caregivers reported persistent, compulsive sugar intake (~100g/day), aversion to salty foods, and behavioral rigidity around meal choices. No mood, psychotic, or obsessive-compulsive symptoms were observed. Findings suggest disruption of right orbitofrontal–insular–limbic connectivity via mass effect, producing a hedonic shift in dietary preference consistent with Gourmand Syndrome.

**Image 1:**
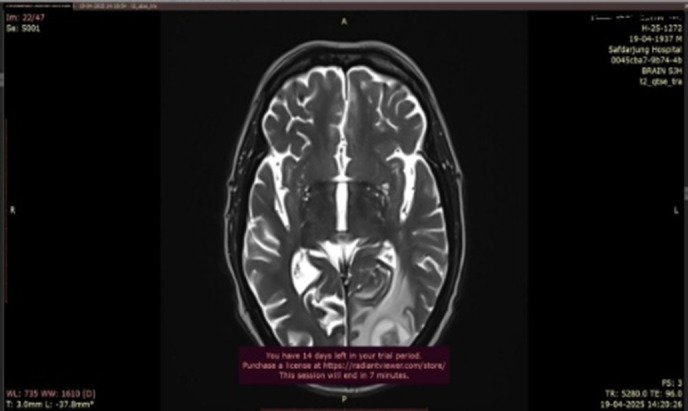
Image 1: Long description.

**Image 2:**
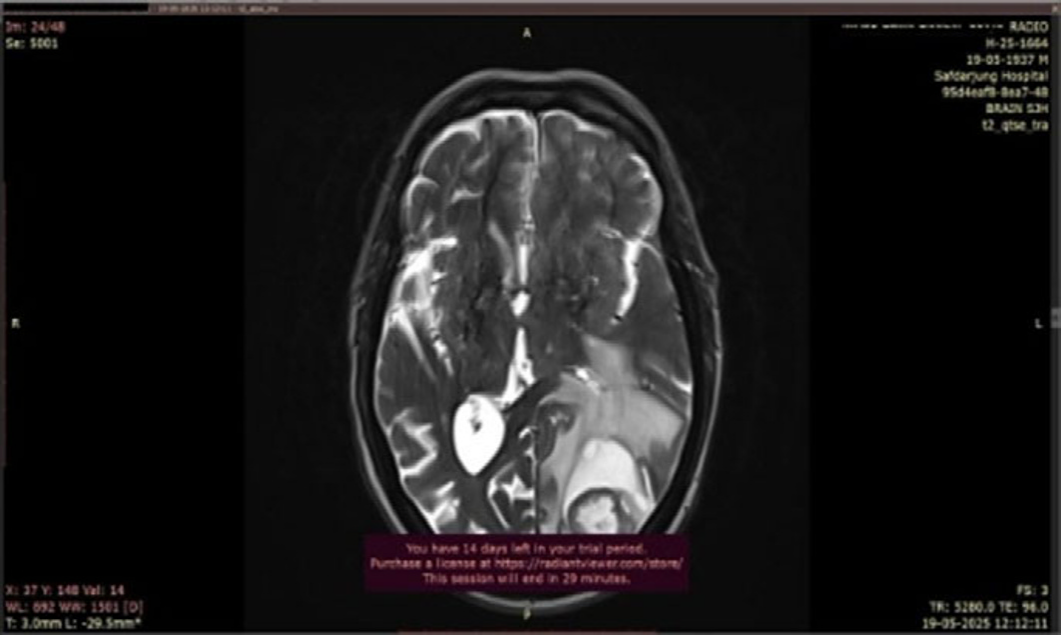
Image 2: Long description.

**Conclusions:**

This case expands the anatomical spectrum of Gourmand Syndrome beyond classic anterior lesions to include posterior metastases with secondary right-sided circuit involvement. Selective dietary changes may serve as clinically valuable behavioral markers of focal brain network disruption. Psychiatric recognition of such presentations is critical for comprehensive management, caregiver counseling, and advancing understanding of brain–behavior relationships.

**Disclosure of Interest:**

None Declared

## Long descriptions

### Image 1: Long description

At the center of the scan are the cerebral ventricles, appearing bright white against the darker brain tissue. The surrounding gray and white matter show the intricate folds of the sulci and gyri. A notable hyperintense area is visible in the posterior region of the right hemisphere. The outermost layer is the dark outline of the skull.

Clinical metadata is distributed in the corners.

Top-left: Im 22 forward slash 47 and Se 5001.

Top-right: Patient information including H dash 25 dash 1272, birth date 19 dash 04 dash 1937 M, Safdarjung Hospital, and scan type B R A I N S I H t 2 underscore qfse underscore tra.

Bottom-left: W L 735 W W 1610 [D] and T 3.0 mm L minus 37.8 mm.

Bottom-right: F S 3, T R 5280.0 T E 96.0, and date 19 dash 04 dash 2025.

A red translucent banner at the bottom center contains trial period text for radiantviewer dot com forward slash store forward slash.

Navigate back to Image 1:.

### Image 2: Long description

This axial cross-section of a human brain is displayed in grayscale. The outer perimeter shows the skull and scalp as a thin dark band. Inside, the cerebral cortex exhibits complex folding with bright white areas indicating cerebrospinal fluid in the sulci and ventricles. A prominent bright white oval shape is visible in the lower left quadrant of the brain tissue.

Metadata is distributed across the four corners of the black background:

* Top Left: Im 24 forward slash 48 and Se 5001.

* Top Right: Patient information including Safdarjung Hospital, BRAIN S I H, and t 2 underscore q t s e underscore t r a.

* Bottom Left: Coordinates X 37 Y 148 Val 14, W L 692 W W 1501, and T 3.0 m m L -29.5 m m.

* Bottom Right: F S 3, T R 5280.0 T E 96.0, and date 19-05-2025.

A purple notification box at the bottom center contains text regarding a trial period for radiantviewer dot com.

Navigate back to Image 2:.

